# Supplementary material for: PgtE protease enables virulent Salmonella to evade C3-mediated serum and neutrophil killing
Source: mBio. 2025 Jul 14;16(8):e03802-24. doi: 10.1128/mbio.03802-24 (PMC12345235; doi:10.1128/mbio.03802-24)
Supplement: Supplemental Material — Supplemental figures and tables. [file mbio.03802-24-s0001.docx]

**Supplemental Figures and Tables**

# PgtE protease enables virulent *Salmonella* to evade C3-mediated serum and neutrophil killing

Michael H. Lee^1^, Araceli Perez-Lopez^1,2^, Leigh A. Knodler^3,4^, Grace Nguyen^1^, Gregory T. Walker^1^, Judith Behnsen^5^, Steven Silva^1^, Jean Celli^3,4^, Melissa A. Tamin^1^, Michael H. Liang^1^, Karine Melchior^1^, Felix A. Argueta^1^, Sean-Paul Nuccio^1^, and Manuela Raffatellu^1,6*^

^1^Division of Host-Microbe Systems and Therapeutics, Department of Pediatrics, University of California San Diego, La Jolla, CA 92093, USA

^2^Biomedicine Research Unit, Facultad de Estudios Superiores Iztacala, Universidad Nacional Autónoma de México. Tlalnepantla, State of México 54090, México

^3^Paul G. Allen School for Global Health, College of Veterinary Medicine, Washington State University, Pullman, Washington, USA

^4^Department of Microbiology and Molecular Genetics, Larner College of Medicine, University of Vermont, Burlington, VT 05405, USA

^5^Department of Microbiology & Immunology, University of Illinois Chicago, Chicago, IL USA

^6^Chiba University-UC San Diego Center for Mucosal Immunology, Allergy, and Vaccines (CU-UCSD cMAV), La Jolla, CA 92093, USA


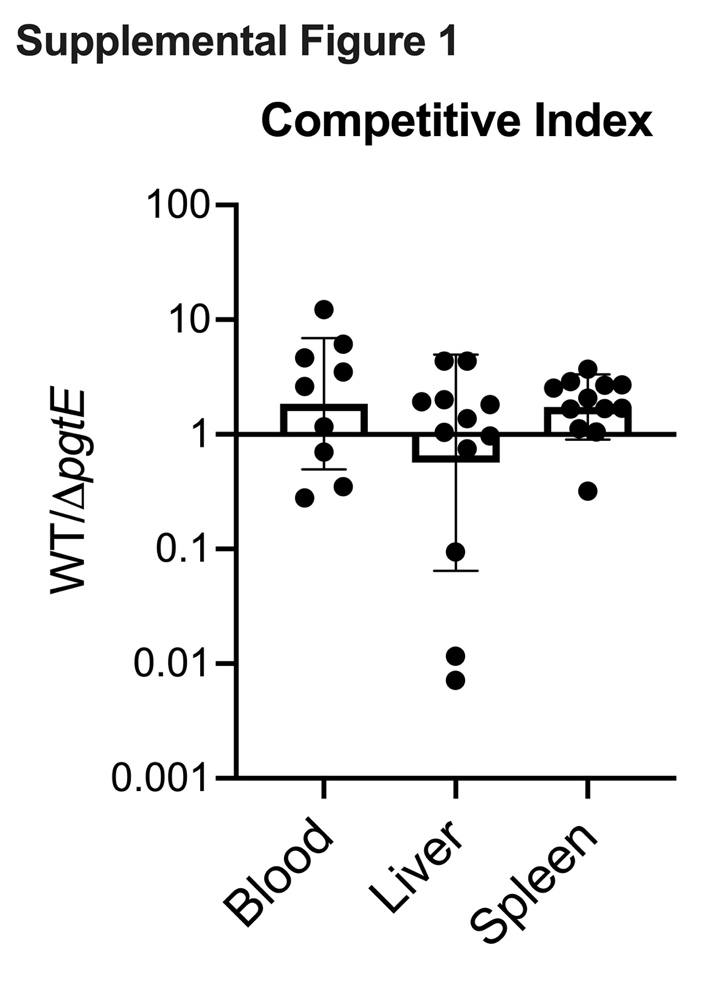


# Supplemental Figure 1. The STm *pgtE* mutant is not attenuated in competitive infections.

8-week-old C57BL/6 wild-type mice were infected intraperitoneally with a 1:1 mixture of STm wild-type (WT, strain IR715) and an isogenic *ΔpgtE* mutant (5x10^3^ CFU each). Bacterial CFUs were enumerated in the blood, liver and spleen at 24 hours post-infection.

The competitive index was calculated by dividing the output CFU ratio (WT divided by *ΔpgtE*) by the input CFU ratio (WT divided by *ΔpgtE*).


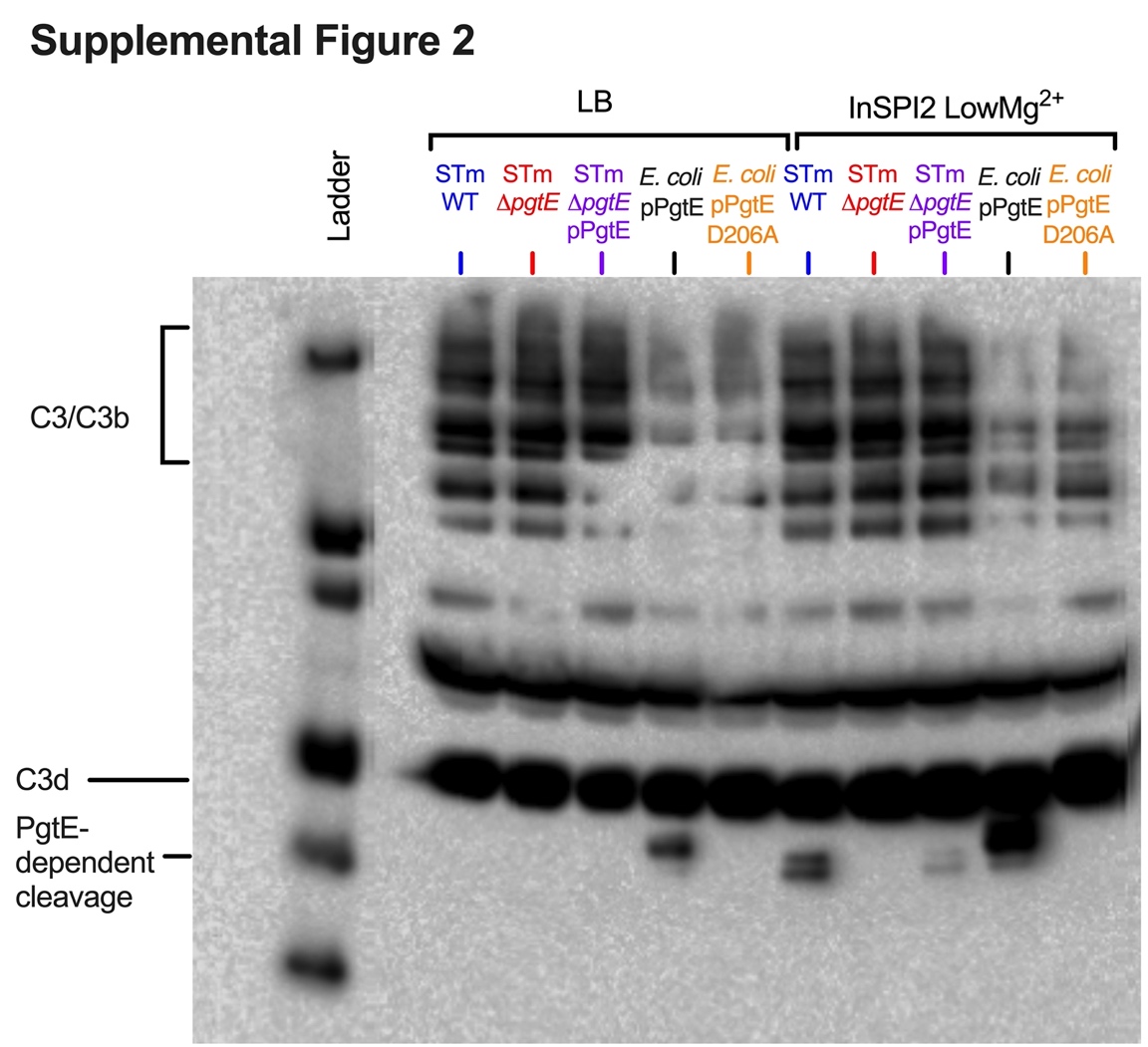


# Supplemental Figure 2. Wild-type STm cleaves complement C3 in a PgtE-dependent manner when grown in conditions that mimic the phagosome

Lane 1 shows the chemiluminescent ladder. Lane 2 is empty. We used the following strains: STm IR715 wild-type (WT), an isogenic *ΔpgtE* mutant, the *ΔpgtE* mutant complemented *in trans* (*ΔpgtE* pPgtE), a rough *E. coli* transformed with a pWSK29 plasmid containing a functional *pgtE* gene (pPgtE*)*, or a *pgtE* gene with a single point mutation inactivating PgtE (pPgtE D206A). The indicated bacterial strains were cultured overnight either in LB (**Left**: Lane 3-7) or InSPI2 LowMg^2+^ minimal media (**Right**: Lane 8-12) before incubation with normal human serum for 8 hours. PgtE-dependent complement cleavage in supernatants was assessed by western blot analysis with an anti-complement C3/C3b/iC3b/C3d antibody.


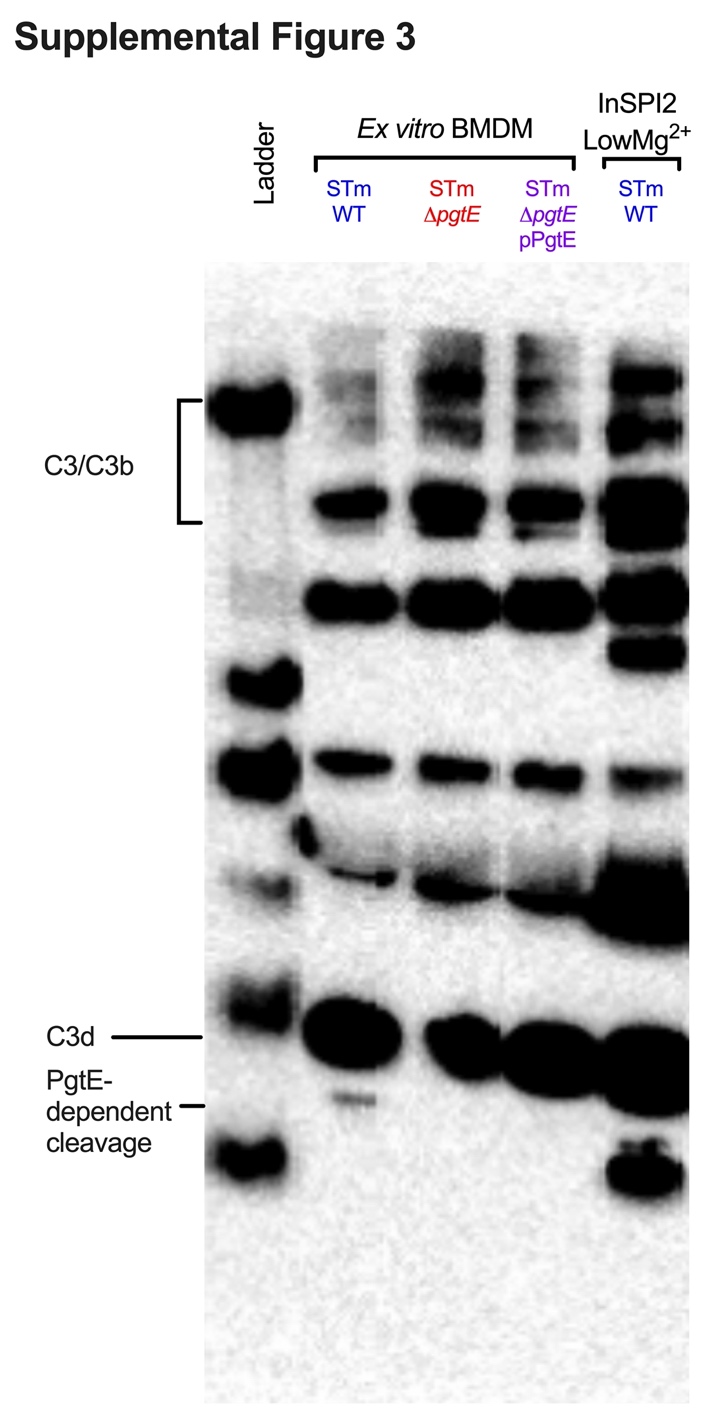


# Supplemental Figure 3. Wild-type STm cleaves complement C3 in a PgtE-dependent manner when grown in macrophages.

Lane 1 shows the chemiluminescent ladder. STm IR715 wild-type (WT: lane 2), the isogenic *pgtE* mutant (*ΔpgtE:* lane 3), and the *ΔpgtE* mutant complemented *in trans* (*ΔpgtE* pPgtE: lane 4) were cultured overnight in LB, then used to infect bone marrow-derived macrophages (BMDMs). STm was isolated from BMDMs 8 hours after infection and then incubated with normal human serum for 13 hours. Alternatively, STm WT was cultured overnight in InSPI2 LowMg^2+^ minimal media and incubated with normal human serum for 13 hours as a control (lane 5). PgtE-dependent complement cleavage in supernatants was assessed by western blot analysis with an anti-complement C3/C3b/iC3b/C3d antibody.


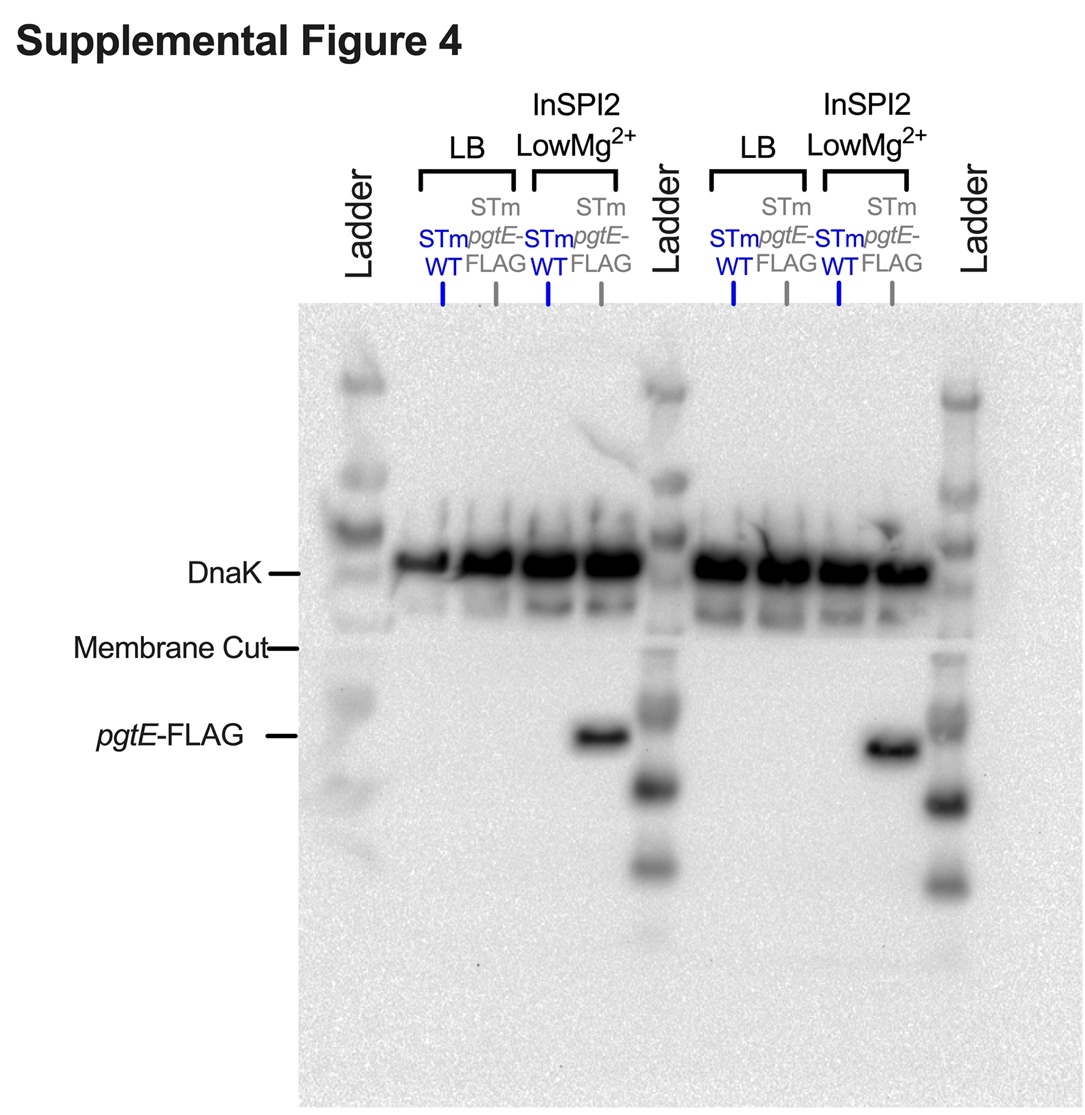


# Supplemental Figure 4. Western blot analysis of STm WT or STm *pgtE*-FLAG cultured overnight in LB or InSPI2 LowMg^2+^ minimal media.

Chemiluminescent ladder is shown in lane 1, 6, and 11. Western blot analysis of STm WT cultured overnight in LB (lane 2, 7) or InSPI2 LowMg^2+^ minimal media (Lane 4, 9) or STm *pgtE*-FLAG cultured overnight in LB (Lane 3, 8) or InSPI2 LowMg^2+^ minimal media (Lane 5, 10). Lane 2-5 and Lane 7-10 are technical replicates. Membrane cut represents where the PVDF membrane was cut prior to staining. The bottom half of the membrane was stained with an anti-FLAG tag antibody. The top half of the membrane was stained with an anti-DnaK antibody as a loading control.


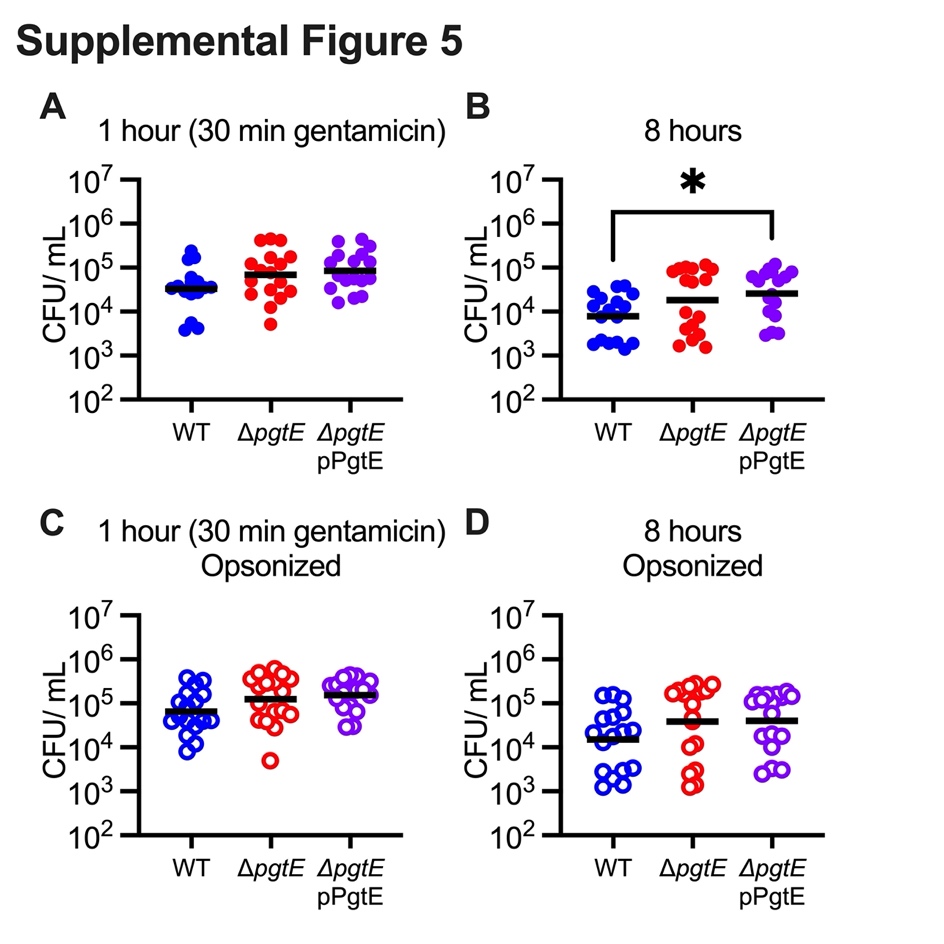


# Supplemental Figure 5. PgtE does not increase STm survival in macrophages under tested conditions.

(**A-D**) BMDMs were infected at an MOI = 1 with IR715 WT, *ΔpgtE*, and *ΔpgtE* pPgtE that were either (**A-B**) not opsonized or (**C-D**) opsonized with normal mouse serum. (**A-D**) 30 minutes after infection, BMDM were then incubated with 100 µg/mL gentamicin for 30 minutes. (**A, B**) For the 1-hour time point, BMDMs were then lysed with 1% Triton-X 100. (**C, D**) For the 8-hour time point, BMDMs were further incubated with 20 µg/mL gentamicin for 7 hours then lysed with 1% Triton-X 100. (**A-D**) N = 17 from 7 independent experiments. Symbols represent data from BMDMs from individual mice, bars represent the geometric means. Data were analyzed by Kruskal-Wallis test (non-parametric, non-paired) followed by Dunn’s multiple comparison test. Adjusted p values from Dunn’s multiple comparison test: * p < 0.05.

#
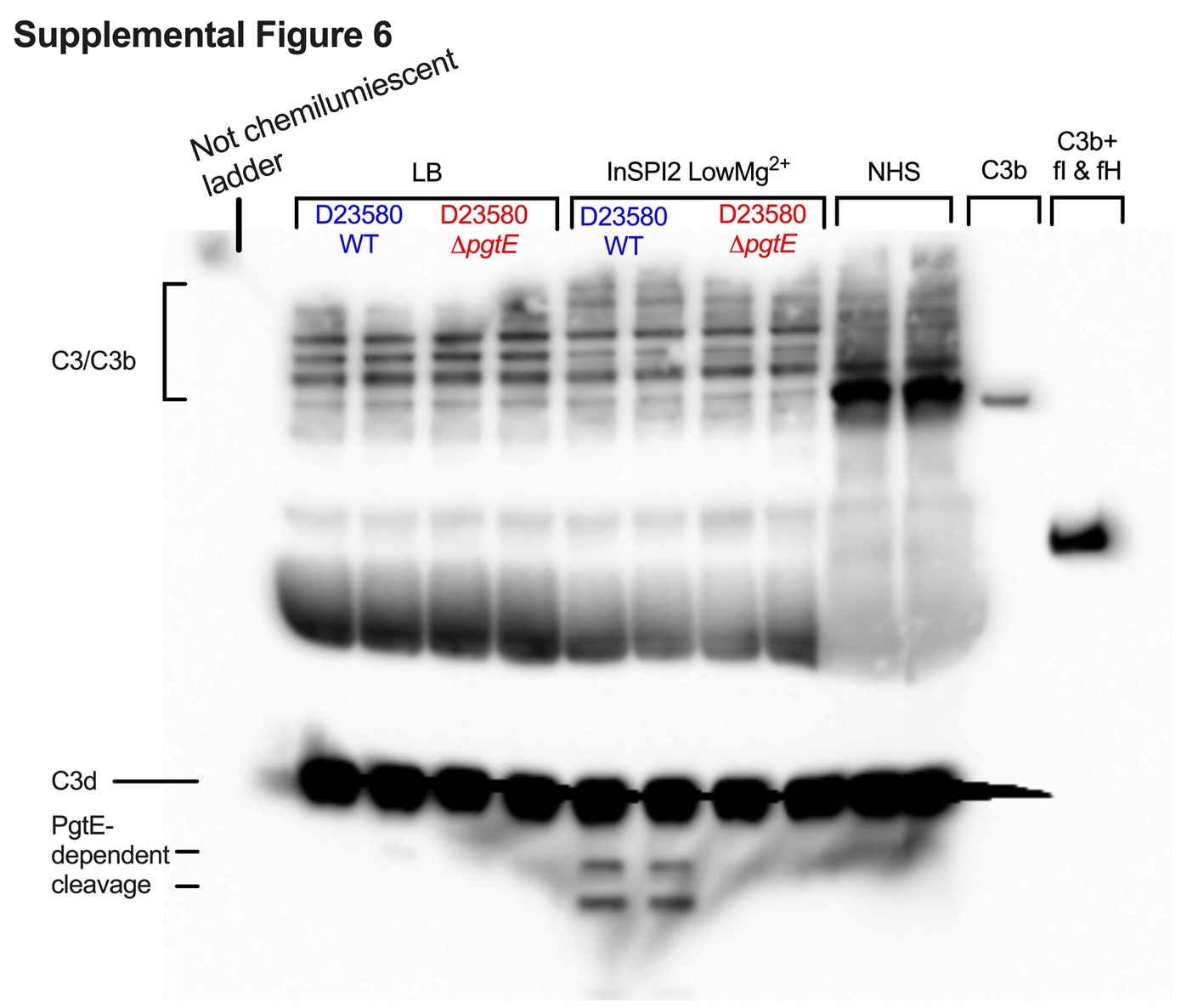
Supplemental Figure 6. iNTS cleaves complement C3 in a PgtE-dependent manner when grown in conditions that mimic the phagosome

Not chemiluminescent ladder is in lane 1. D23580 WT (Lane 2, 3) and an isogenic *ΔpgtE* mutant (Lane 4, 5) cultured overnight in LB. D23580 WT (Lane 6, 7) and *ΔpgtE* (Lane 8, 9) cultured overnight in InSPI2 LowMg^2+^ minimal media. STm was then incubated with normal human serum (NHS) for 8 hours. Alternatively, NHS was incubated without *Salmonella* for 8 hours (Lane 10, 11). In the last two lanes, purified complement C3b was incubated alone (Lane 12) or with complement cofactor I and cofactor H (fI & fH: Lane 13). PgtE-dependent complement cleavage in supernatants was assessed by western blot analysis with an anti-complement C3/C3b/iC3b/C3d antibody.


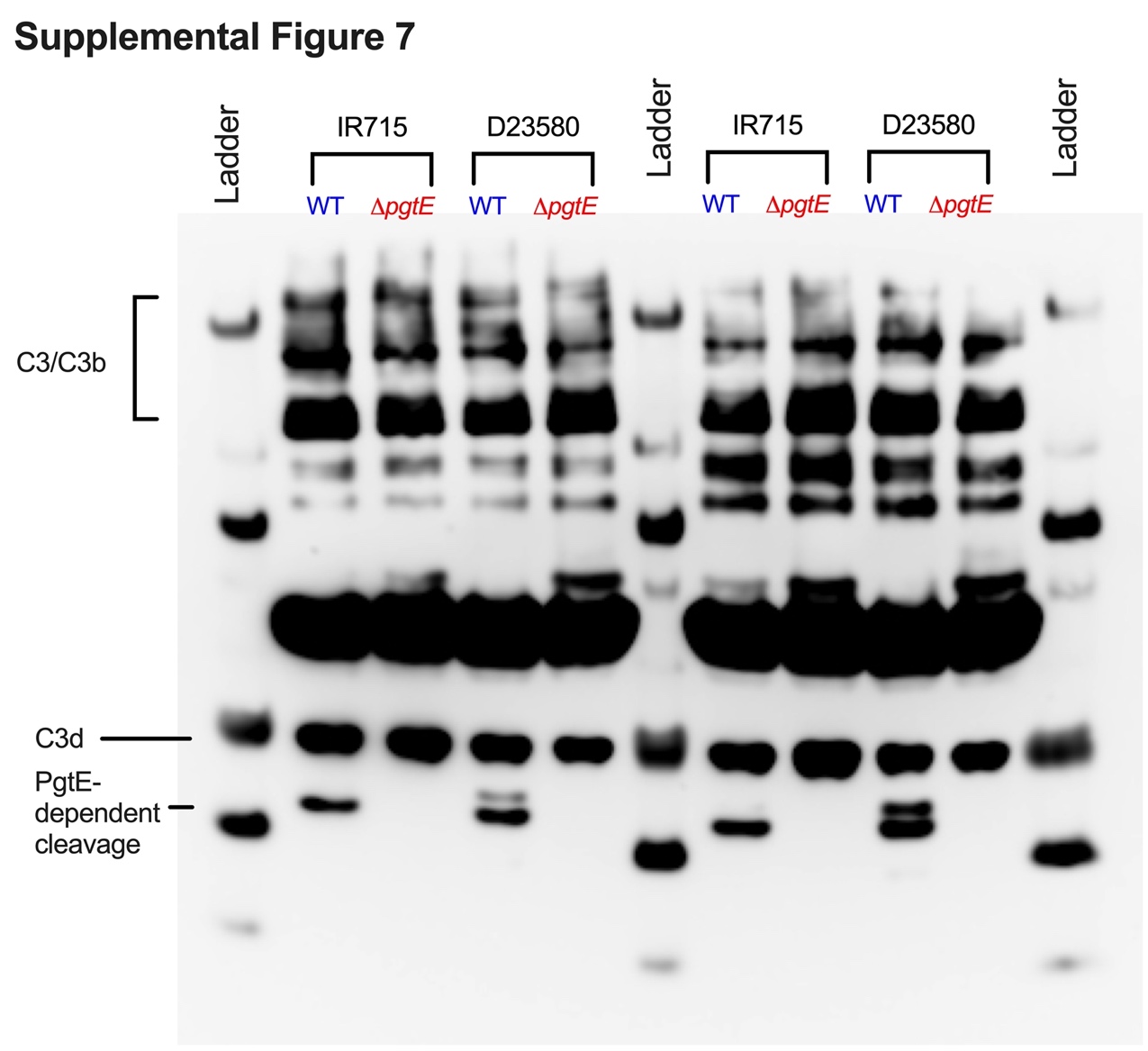


# Supplemental Figure 7. iNTS strain D23580 cleaves complement C3 more than NTS strain IR715 when grown in conditions that mimic the phagosome

Chemiluminescent ladder is in lane 1, 6, and 11. IR715 WT (Lane 2, 7) and the *ΔpgtE* mutant (Lane 3, 8) and D23580 WT (Lane 4, 9) and *ΔpgtE* (Lane 5, 10) were cultured overnight in InSPI2 LowMg^2+^ minimal media. STm strains were then incubated with NHS for 8 hours. PgtE-dependent complement cleavage in supernatants was assessed by western blot analysis with anti-complement C3/C3b/iC3b/C3d antibody.

# Supplemental Table 1

Strains used in this study

| **Designation** | **Genotype** | **Reference or Source** |
| --- | --- | --- |
| ***Salmonella enterica* serovar Typhimurium** | | |
| IR715 | ATCC 14028s wild-type, spontaneous Nal^R^ | Stojiljkovic et al, J. Bacteriol. 177(5):1357-1366 (1995) |
| JB10 | IR715 Δ*pgtE*::*tetRA* (Nal^R^, Tet^R^) | This study |
| ML27 | IR715 *pgtE*-FLAG (Nal^R^) | This study |
| LAKglmS | *SL1344 glmS::Ptrc-mCherryST::FCF (Strep^R^, Cm^R^)* | Knodler et al, Cell Host Microbe. 16(2):249-256 (2014) |
| LAKML2 | IR715 *glmS*::Ptrc-mCherrryST::FRT (Nal^R^) | This study |
| D23580 | D23580 wild-type | Kingsley et al, Genome Research. 19:2279–2287 (2009) |
| SPN1113 | D23580 Δ*pgtE*::*tetRA* | This study |
| ***Escherichia coli*** |  |  |
| CC118 λ*pir* | F- *araD139* Δ(*ara*, *leu*)7697 Δ*lacX74* *phoA*D20 *galE* *galK* *thi rpsE* *rpoB* *argE*^am^ *recA1* λ*_pir_* | Herrero et al, J Bacteriol. 172(11):6557-67 (1990) |
| S17-1 λ*pir* | F- *recA* *thi pro* rK- mK+ RP4:2-Tc::*Mu*Km Tn7 λ*_pir_* | Herrero et al, J Bacteriol. 172(11):6557-67 (1990) |
| XL1-Blue | *recA1* *endA1* *gyrA96* *thi-1* *hsdR17* *supE44* *relA1* *lac* [F *proAB* *lacI^q^Z*ΔM15 Tn10 (Tet^R^)] | Agilent |
| DH5αMCR | F- *mcrA* Δ(*mrr-hsdRMS-mcrBC*) φ80d*lacZ*ΔM15 Δ(*lacZYA-argF*)U169 *deoR recA1 endA1 phoA supE44*λ- *thi-1 gyrA*96 *relA1* | Gibco BRL |
| One Shot TOP10 | F- *mcr*A Δ(*mrr-hsdRMS*-*mcrBC*) φ80*lacZ*ΔM15 Δ*lacX74* *recA1* *araD139* Δ(*ara, leu*)7697 *galU* *galK* *rpsL*(Str^R^) *endA1* *nupG* | Invitrogen |

# Supplemental Table 2

Plasmids used in this study

| **Designation** | **Relevant characteristics** | **Reference or Source** |
| --- | --- | --- |
| pCP20 | Ap^R^, temperature-sensitive, FLP recombinase system | Datsenko et al, Proc. Natl. Acad. Sci. USA. 97, 6640-6645 (2000) |
| pWSK29 | Ap^R^, MCS, *lacZa* | Wang et al, Gene. 100, 195-199 (1991) |
| pWSK29::*pgtE* | Ap^R^ Tet^R^, pWSK29::*pgtE* (*pgtE* complementation) | This study |
| pWSK29::*pgtE*-D206A | Ap^R^ Tet^R^, pWSK29::*pgtE*(D206A) (PgtE inactive allele) | This study |
| pRDH10 | Cm^R^ Tet^R^, SacB (levansucrase: Sucrose sensitivity) | Kingsley et al, Applied and Environmental Microbiology, 1610-1618 (1999) |
| pRDH10*::pgtE-FLAG* | Cm^R^ Tet^S^, SacB, pRDH10*::pgtE-FLAG* (pgtE-FLAG Tag) | This study |
| pCR-Blunt II-TOPO | Kan^R^, MCS | Invitrogen |
| pGP704 | Ap^R^, MCS, *oriR6K*, *mobRP4* | Miller et al, J. Bacteriology. 170(6):2575-2583 (1988) |
| pSPN23 | Ap^R^ Tet^R^, pBluescriptII KS+::*tetRA* (*tetRA* cassette) | Raffatellu et al, Cell Host Microbe. 5(5):476-86 (2009) |
| pCRII*::pgtE-*LBRB | Kan^R^, pCR-Blunt II-TOPO::*pgtE*-LBRB (Δ*pgtE* cassette) | This study |
| pGP704*::pgtE-*LBRB | Ap^R^, pGP704::*pgtE*-LBRB (Δ*pgtE* cassette) | This study |
| pGP704*::pgtE*-LBRB*::tetRA* | Ap^R^ Tet^R^, pGP704_pgtE_LBRB::*tetRA* (Δ*pgtE*::*tetRA* cassette) | This study |
| pP*_pgtE_-gfp* | Ap^R^*,* P*_pgtE_-gfpmut3.1* (*pgtE* transcriptional reporter plasmid) | This study |
| pMPM-A3∆Plac | Ap^R^, P15A ori | Ibarra et al., Microbiology Apr;156(Pt 4):1120-1133 (2010) |

# Supplemental Table 3

Primers used in this study

| **Designation** | **Purpose** | **Primer sequence (5' to 3')** | | **Reference or Source** |
| --- | --- | --- | --- | --- |
| pgtE_LB_for | Amplifying *pgtE* upstream region | ATCAGCAGAGATCATCATGG | | This study |
| pgtE_RB_rev | Amplifying *pgtE* downstream region | AATTGAAGACGCGCTACG | | This study |
| pgtE_LB_r_fus* | pCRII_*pgtE*_LBRB fusion | TGACAAGATGGCTTCTAGACCACATCGG | | This study |
| pgtE_RB_f_fus * |  | GTCTAGAAGCCATCTTGTCAAATCGTCGG | | This study |
| pgtE_LB_f_SalI* | pWSK29_*pgtE*_compl | GTCGACAATCTCGGCTATACCTTTGG | | This study |
| pgtE_RB_r_EcoRO* |  | GATTCCCGTTATCTCCATCAACTGG | | This study |
| pgtE_RB_r_seq | pCRII_pgtE_LBRB sequencing | CGTTGAAGAGTATGAGCGAC | | This study |
| pgtE_pres_for | Colony PCR screening | CACCGCTGGTTTTATCTATG | | This study |
| pgtE_pres_rev |  | ACGTCTCTCCTGATAGCGTC | | This study |
| tetRA_pres_for | PCR confirmation of *tetRA* cassette presence | TTCGGAAGATATCGCTAACC | | This study |
| tetRA_pres_rev |  | TAAAGCACCTTGCTGATGAC | | This study |
| tetR_int_rev | *tetRA* cassette presence | CAGAGCCAGCCTTCTTATTC | | This study |
| tetA_int_for |  | GATGACCTTCATGTTAACCC | | This study |
| pgtE_for_compl | *pgtE* complementation | TTATGACCGATGACATCCC | | This study |
| pgtE_rev_compl |  | AATGCGTCAAGTTCTCTGG | | This study |
| PpgtE-XbaI-F* | *pgtE* transcriptional reporter plasmid | GCTCTAGAACGAATTAATGAAAGTGGC | | This study |
| PpgtE-SmaI-R* |  | TCCCCCGGGATCATCATTACTGCAATAGCA | | This study |
| FLAG_Upstream_Fwd** | Amplify upstream of *pgtE* stop codon for FLAG tag Gibson assembly | gggcgccatctccttgcatgACAAGGCGGGCGTAACAG | | This study |
| FLAG_Upstream_Rev*** |  | cttgtcatcgtcgtccttgtagtcGAAGCGATACTGCAACCCC | | This study |
| FLAG_Downstream_Fwd*** | Amplify *pgtE* stop codon and downstream for FLAG tag Gibson assembly | gactacaaggacgacgatgacaagTAGACCACATCGGGATGTC | | This study |
| FLAG_Downstream_Rev** |  | ggccatccagcctcgcgtcgCCTGGAGCGACTTTCTCTG | | This study |
| FLAG_Verification_Fwd | Verify clean insertion of FLAG tag in *pgtE* | TTCCGGACGTCTCTCCTGAT | | This study |
| FLAG_Verification_Rev |  | ACGCGATTATCTCTGGCTGG | | This study |
| pgtE F1 | *pgtE* qPCR | CACGGAAGGTAAGTTTGCG | | This study |
| pgtE B1 |  | ACAGCGACTGGGTAAATGC | | This study |
| 16s_Fwd | *16s* qPCR | TGTTGTGGTTAATAACCGCA | | Barman et. al. Infect Immun  . 2008 Mar;76(3):907-15 |
| 16s_Rev |  | GACTACCAGGGTATCTAATCC | |  |
| * = restriction sites are underlined | | |  |  |
| ** = engineered sequence for pRDH10 homology are underlined | | |  |  |
| *** = engineered sequence for FLAG Tag are underlined | | |  |  |
